# COVID-19 and Acute Pulmonary Embolism in Postpartum Patient

**DOI:** 10.3201/eid2608.201383

**Published:** 2020-08

**Authors:** Zohre Khodamoradi, Shahrokh Sadeghi Boogar, Farnaz Kamali Haghighi Shirazi, Pariya Kouhi

**Affiliations:** Shiraz University of Medical Sciences, Shiraz, Iran

**Keywords:** Coronavirus diseases, 2019 novel coronavirus disease, COVID-19, SARS-CoV-2, severe acute respiratory syndrome coronavirus 2, respiratory diseases, zoonoses, viruses, Iran, pneumonia, acute pulmonary embolism, postpartum, Iran

## Abstract

We report a 36-year-old woman in Iran who sought care for left shoulder pain and cough 5 days after a scheduled cesarean section. Acute pulmonary embolism and coronavirus disease were diagnosed. Physicians should be aware of the potential for these concurrent conditions in postpartum women.

An outbreak of viral pneumonia that emerged in late 2019 and spread rapidly worldwide was named coronavirus disease (COVID-19) (*1*). COVID-19 is caused by severe acute respiratory syndrome coronavirus 2 (SARS-CoV-2). Two other viruses of this family, severe acute respiratory syndrome coronavirus and Middle East respiratory syndrome coronavirus, also have caused outbreaks globally (*1*).

Venous embolism has been associated with severe infection. Acute pulmonary embolism has been associated with severe acute respiratory syndrome coronavirus infections, but no cases have been reported with Middle East respiratory syndrome (*2*,*3*). A study reported a 75-year-old hospitalized woman with COVID-19 and pulmonary embolism (*4*). In addition, in 2 COVID-19–positive patients, 57 and 70 years of age, from Wuhan, China, computed tomography angiography (CTA) confirmed pulmonary embolism (*5*). Three cases of deep vein thrombosis with COVID-19 also have been reported (*6*).

Pregnancy increases the risk for venous embolism (*7*). Although approximately half of venous embolism occurs during pregnancy and half occurs during the postpartum period, the risk per day is greatest in the weeks immediately after delivery (*8*). We report a patient in Iran who sought care for cough and shoulder pain 5 days after an uncomplicated cesarean delivery in whom an acute pulmonary embolism and COVID-19 infection were subsequently diagnosed. The ethics committee of Shiraz University of Medical Sciences (Shiraz, Iran) approved the study.

A healthy 36-year-old nonsmoking woman (gravid 2, 1 term infant delivered, 1 abortion/miscarriage) underwent an elective scheduled caesarean section at 37 weeks 2 days of gestation after an uncomplicated pregnancy. The uncomplicated surgery resulted in the birth of a healthy infant. Mechanical prophylaxis to prevent deep vein thrombosis was used at delivery until ambulation. The woman was discharged on postpartum day 2 in a good condition. On postpartum day 5, she sought care for sudden onset left-side shoulder pain and dry cough. She stated that she did not have fever, myalgia, or diarrhea. On postpartum day 5, she experienced mild shortness of breath. During her pregnancy, she had no known history of contact with persons who had confirmed or suspected COVID-19.

At admission, physical examination revealed a blood pressure of 110/70 mm Hg, body temperature of 36.8°C, pulse rate of 92 beats/min, respiratory rate of 20 breaths/min, and oxygen saturation of 94% on ambient air. Her body mass index was 24.8 kg/cm^2^. Her physical examination was otherwise unremarkable.

Laboratory test results showed a complete blood count and leukocyte differentials within reference ranges but elevated liver function tests, C-reactive protein level, and erythrocyte sedimentation rate. D-dimer was 800 μg/mL (reference <500 μg/mL). Results of her baseline electrocardiogram were unremarkable. She had a normal echocardiography with ejection fraction of »60%.

Because of the COVID-19 pandemic and the patient’s report of cough, she underwent screening for SARS-CoV-2. Throat swab samples were positive for SARS-CoV-2 by real-time reverse transcription PCR. Moreover, because of her clinical features, history, risk for venous embolism, and high level of D-dimer, CTA was performed. Thoracic CTA on the first day of hospitalization showed emboli in the right side interlobar artery, posterior basal segment, and the lingular branch (Figure, panels A, B). Hampton hump in the right side posterior basal segment was consistent with lung infarction. CTA further revealed left-sided pleural effusion associated with new mixed consolidation and ground glass opacifications (Figure, panels C, D).

**Figure:**
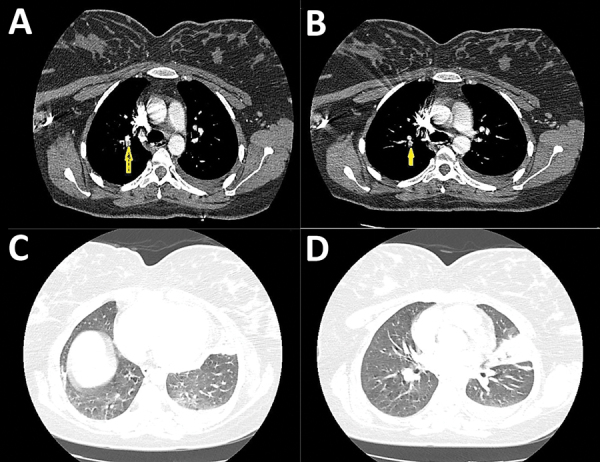
Thoracic computed tomography angiography of a 36-year-old postpartum woman with coronavirus disease and acute pulmonary embolism, Iran. A, B), Thoracic computed tomography angiography showing filling defect in the right side inter-lobar artery (arrow) and posterior basal segment and left-sided pleural effusion (arrow). C, D) Consolidation and ground-glass opacities affecting the left ligula and posterior recess.

CTA findings were consistent with pneumonia, pulmonary embolism, and lung infarction. The patient was treated with enoxaparin (1 mg/kg subcutaneously 2×/d). She was discharged in good condition with enoxaparin for 6 months.

Multiple conditions made this patient susceptible to pulmonary embolism. Because inflammation and coagulation are related, infected patients have hypercoagulable state (*2*). Virchow’s triad, which contributes to thrombosis, has 3 factors: venous stasis, hypercoagulability, and endothelial injury. Septic patients have criteria of Virchow’s triad; cesarean section as a surgery contributed to Virchow’s triad in this patienet because endothelial injury made the patient prone to embolic events (*7*–*9*).

The patient we report was young, was not critically ill or septic, and had no evidence of disseminated intravascular coagulation. Alteration in coagulation pathways during pregnancy increases the risk for embolic events. The risk in the immediate postpartum period is particularly high. Venous embolism is an important cause of maternal illness and death (*7*).

CTA or ultrasonography for deep vein thrombosis may be important for COVID-19–positive pregnant or postpartum patients who have signs or symptoms of possible venous embolism, given their potentially heightened risk. In this patient population, with an already elevated risk for venous embolism, physicians should be aware of the potential for concurrent mild COVID-19 and acute pulmonary embolism.
